# Repeat-aware modeling and correction of short read errors

**DOI:** 10.1186/1471-2105-12-S1-S52

**Published:** 2011-02-15

**Authors:** Xiao Yang, Srinivas Aluru, Karin S Dorman

**Affiliations:** 1 Department of Electrical and Computer Engineering, Iowa State University, Ames, Iowa, 50011, USA; 2 Department of Computer Science and Engineering, Indian Institute of Technology Bombay, Mumbai, Maharashtra, 400 076, India; 3 Department of Statistics and Department of Genetics, Development & Cell Biology, Iowa State University, Ames, Iowa, 50011, USA

## Abstract

**Background:**

High-throughput short read sequencing is revolutionizing genomics and systems biology research by enabling cost-effective deep coverage sequencing of genomes and transcriptomes. Error detection and correction are crucial to many short read sequencing applications including *de novo* genome sequencing, genome resequencing, and digital gene expression analysis. Short read error detection is typically carried out by counting the observed frequencies of *k*mers in reads and validating those with frequencies exceeding a threshold. In case of genomes with high repeat content, an erroneous *k*mer may be frequently observed if it has few nucleotide differences with valid *k*mers with multiple occurrences in the genome. Error detection and correction were mostly applied to genomes with low repeat content and this remains a challenging problem for genomes with high repeat content.

**Results:**

We develop a statistical model and a computational method for error detection and correction in the presence of genomic repeats. We propose a method to infer genomic frequencies of *k*mers from their observed frequencies by analyzing the misread relationships among observed *k*mers. We also propose a method to estimate the threshold useful for validating *k*mers whose estimated genomic frequency exceeds the threshold. We demonstrate that superior error detection is achieved using these methods. Furthermore, we break away from the common assumption of uniformly distributed errors within a read, and provide a framework to model position-dependent error occurrence frequencies common to many short read platforms. Lastly, we achieve better error correction in genomes with high repeat content. *Availability*: The software is implemented in C++ and is freely available under GNU GPL3 license and Boost Software V1.0 license at “http://aluru-sun.ece.iastate.edu/doku.php?id=redeem”.

**Conclusions:**

We introduce a statistical framework to model sequencing errors in next-generation reads, which led to promising results in detecting and correcting errors for genomes with high repeat content.

## Background

High throughput next generation sequencing has revolutionized genomics, making it possible to sequence new genomes or resequence individual genomes at a manifold cheaper cost and in an order of magnitude less time than earlier Sanger sequencing. With this technology, ambitious genome sequencing projects target many organisms rather than a few, and large scale studies of sequence variation become feasible [1]. Many next-geneneration sequencing technologies have been developed, including systems currently in wide use, such as the Illumina Genome Analyzer (earlier known as Solexa) and Applied Biosystems SOLiD, as well as more recent and new offerings from companies such as Complete Genomics and Pacific Biosciences [2]. Many next-generation sequencing systems produce short reads, e.g., the widely used Illumina Genome Analyzer systems typically produce 35 - 150bp reads. Short read technologies have been widely adopted for both genome sequencing and resequencing applications; hence, development of high quality short read assemblers (e.g., [3-7]) and short read mapping tools that map reads to a reference genome [8,9] are important.

Short reads of novel genomes are typically assembled using de Bruijn graphs that represent observed *k*mers as nodes and length (*k* – 1) overlaps as edges. In the absence of errors, the size of such a graph is bounded by the length of the genome, but can be as high as 4*^k^* in the presence of errors. In the mapping process, a read with sequencing errors may map to multiple locations, or sometimes nowhere at all. Thus, error removal or correction is necessary to keep the size of the graph manageable [7,10] and simplify non-repetitive read mapping [11].

Many approaches have been proposed to identify and sometimes correct sequencing errors in next-generation sequencing data. More recent ones include SAP (Spectral Alignment Problem)-based methods [4,10], SHREC [12] and Reptile [13]. SAP-based methods identify any *k*mer occurring less than a constant, user-specified frequency threshold to be erroneous. Chin *et al*. [14] have shown that an optimum threshold can be derived analytically, assuming the genome to be a random sequence and that errors are independently and uniformly distributed in the reads. SHREC, a suffix trie based method, classifies any substring that occurs less often than an analytically calculated threshold to contain errors based on the same assumptions as in [14]. An erroneous base, identified as an infrequent branch of the suffix trie, is corrected to one of its siblings when applicable. Reptile explores read decompositions and makes corrections to any substring whenever an unambiguous choice can be made. In contrast to the previous two approaches, erroneous substrings are inferred based on assessing their frequencies relative to the frequencies of the alternative (error free) substrings. This accommodates under-sampled genomic regions. All of these methods are mainly suitable for genomes with a low degree of repetitive sequences.

Repeats in genomes can lead to mishandling of errors in many ways. Nearly identical repeats can easily be mistaken to be sequencing errors. Even when errors are rare, an erroneous *k*mer may appear at a moderate frequency if it has few nucleotide differences from one or more valid *k*mers that have a high frequency of occurrence in the genome. The problem of detecting and correcting sequencing errors among reads in the presence of repeats has so far not been adequately addressed. Nevertheless, repeats are widely prevalent, even in some viral genomes such as *N. meningitidis*. Other genomes, like those of plants, are known for their high repeat content; for instance, an estimated 65-80% of the maize genome is spanned by repeats, which makes the assembly, mapping and error detection and correction tasks difficult. Although packages like FreClu [11] and Recount [15] could be potentially adapted to consider repeats, they are specifically designed for transcriptome data and correct read counts rather than identify and correct erroneous bases within reads. Moreover, insufficient replication of full length reads in genomic data prevents these methods from accurately estimating model parameters.

In this paper, we address the problem of identifying and correcting sequencing errors in short reads from genomes with different levels of repetition, particularly for reads produced by the widely used Illumina Genome Analyzer platform. Similar to existing approaches, we decompose the input reads into *k*mer substrings and count the number of times *Y_l_* each *k*mer *x_l_* occurs in the reads. However, instead of inferring erroneous *k*mers based on these observed occurrence frequencies [10,12], we developed a maximum likelihood estimate of the expected number *T_l_* of *attempts* to read *x_l_*, including both attempts that resulted in error-free reads and erroneous reads. In addition, we propose a new method to choose the threshold, which can be used to identify erroneous *k*mers as those *x_l_*’s for which *T_l_*’s are lower than the threshold. We demonstrate that using estimates of read attempts enables more accurate detection of sequencing errors than using observed frequencies for a wide choice of thresholds. We further develop an error correction method to transform erroneous bases in each read to the correct ones and compare the results with SHREC [12] and Reptile [13], two of the most recent error correction methods. The results demonstrate significant improvement in error correction capabilities for genomes with high repeat content. The proposed method is made available through the software package REDEEM (Read Error DEtection and Correction via Expectation Maximization) at “http://aluru-sun.ece.iastate.edu/doku.php?id=redeem”.

## Methods

Let *G* denote the reference genome to be sequenced, and let *R* = {*r*_1_, *r*_2_,…, *r_N_*} be the collection of resulting short reads. For simplicity, we assume each read has a fixed length *L.* The sequence coverage is  , where |*G*| is the genome length. Define the *k*-spectrum of a read *r* to be the set *r^k^* = {*r*[*i : i* + *k* – 1] | 0 ≤ i <*L* – *k* + 1}, where *r*[*i* : *j*] is the substring from position *i* to *j* in *r.* The *k*-spectrum produced by all the reads is .

**Figure 1:**
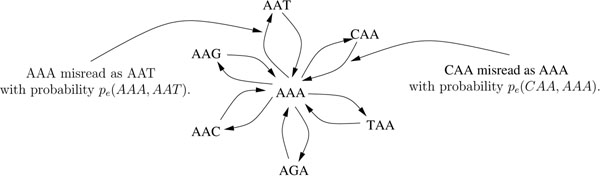
**A *k*mer neighborhood.** The neighborhood of trimer AAA is the collection of trimers in *R*^3^ that have a nonvanishing chance of being misread as AAA, in this case trimers with at most one substitution.

Each *k*mer *x_l_* ∈ *R^k^* has *α_l_* occurrences in *G* and *Y_l_* instances observed in read set *R.* Define  , the probability that a random *k*-length fragment in the genome is *k*mer *x_l_.* Occurrence frequency *α_l_*, or equivalently *s_l_*, is unobserved, but of paramount interest. Indeed, if we knew *s_l_* = 0, but observed *Y_l_* > 0, then we would know each observed instance of *x_l_* contains at least one misread base. Under the assumption that errors are rare, it makes sense to label *k*mers *x_l_* with *Y_l_* <*M* as errors, where *M* is chosen such that *P*(*Y_l_* <*M | s_l_* > 0) is reasonably small. Since *s_l_* is unknown, the threshold *M* is set *ad hoc*, based on training or simulated data [10], or analytical calculations [12,14] assuming the genome to be a random sequence and errors to be distributed uniformly in the reads. In practice, these assumptions do not hold true. Moreover, the problem of misread *k*mers contributing to the observed frequencies *Y_l_* (see Fig. 1) is exacerbated in repetitious genomes where *k*mers with high genomic occurrence may result in generation of the same misread multiple times [10].

We will develop a model that estimates the expected number of *attempts T_l_* to read each *k*mer *x_l_.* The threshold is then applied to each estimated *T_l_* instead of the corresponding observed *Y_l_.* The model we propose is similar to that of RECOUNT [15] used to correct next generation short read counts. Both models derive from a method originally meant to detect sequencing errors in SAGE libraries [16]. Our model differs from the previous models in that it works with *k*mers rather than full reads, since there is insufficient replication of full length reads in genomic data (as compared to transcriptome data). In addition, instead of assuming the misread bases to be drawn from {A, C, G, T} with equal probability, we propose a parametric error model that can be trained from the reads produced by the control lane (e.g.using the Illumina Genome Analyzer) in the same experiment. This strategy has already proven to be useful in several pioneering works [11,17]. In addition, we will show that the model is somewhat robust to incorrect assumptions in the underlying error model. A further step is to modify erroneous bases to their true forms in each read. This task has rarely been attempted previously for repetitive regions. We propose a method that utilizes transition probabilities and the contexual information of individual reads to achieve this goal. Like others, we ignore insertion and deletion errors assuming they are rarely produced by next-generation sequencing technology, which is true for reads from the Illumina Genome Analyzer [18].

### Error model

The simplest error model posits that sequencing errors occur independently at all sites with constant probability *p_e_.* Let *p_e_*(*x_m_*,*x_l_*) be the probability that *x_m_* is misread as *x_l_.* This model produces symmetric misread probabilities:(1)

where *d*(·, ·) denotes the Hamming distance between two *k*mers. It is known, however, that short read technology produces errors with distinct patterns [18]. As a first approximation, we assume that errors strike sites in the *k*mer independently, but with varying probabilities. For example, we observe in dataset preparation section that errors cluster in the 3′ portion of reads and, consequently, *k*mers. Let *q_i_*(*α*,*β*) be the probability that nucleotide *α* at position *i* of a *k*mer is (mis)read as nucleotide *β*, with ∑*_β_ q_i_*(*α*, *β*) = 1. Then, the misread probability is

These misread probabilities are no longer symmetric, and can be arranged into a 4*^k^* × 4*^k^* matrix *P_e_*, where non-zero entries in the *l*th row identify all possible ways to (mis) read *k*mer *x_l_.*

We now discuss some ways to reduce and simplify the calculations. We observe substitution errors are relatively rare, so misread *k*mers generally contain far fewer than *k* errors. Thus, when considering possible origins of a misread *k*mer, we can safely restrict our attention to *k*mers within some Hamming distance *d*_max_ from the current *k*mer. Capping the maximum distance between *k*mers at *d*_max_ induces a sparse *P_e_*, whose entries are normalized by dividing each row by the corresponding row sum. Finally, we ignore *k*mers that are not observed in the data (i.e. *Y_m_* = 0 or, equivalently, *x_m_* ∉ *R^k^*), so the (incomplete) neighborhood of *k*mer *x_l_*, denoted by  , is given as {*x_m_* ∈ *R^k^* : *d*(*x_l_*,*x_m_*) ≤ *d*_max_}*.* Failure to include unobserved *k*mers could bias estimation of *α_l_* by ignoring *k*mers actually present in *G* and capable of contributing to *Y_l_.* However, the bias cannot be large since *α_m_* must be small, otherwise *Y_m_* would not be zero.

After considering errors and applying the simplifications, the counts *Y_l_* follow a Multinomial distribution

***Y*** = (*Y*_1_,…,*Y_|R^k^|_*) ~ Multinomial(*N*(*L* – *k* + 1),***p***),

but unobserved *k*mers are ignored and the probability vector ***p*** = (*p*_1_,*p*_2_ …,*p_l_* …,*p_|R^k^|_*) depends on the *k*mer neighborhood. Specifically,

where ***s*** = (*s*_1_,…,*s_|R^k^|_*) is restricted to the set of observed *k*mers *R^k^.* It becomes clear that when *x_l_* is surrounded by highly repetitious *x_m_* with large *s_m_*, then *Y_l_* may exceed threshold *M* because of high misread occurrence with probability *s_m_p_e_*(*x_m_*,*x_l_*)*.* Thus, when errors combine with repeats, it is more appropriate to apply a threshold to estimates of the parameters *s_l_* than observed *Y_l_.*

The observed log likelihood, *l*(*s |****Y***), involves a mixture over the neighborhood (Fig. 1) of *k*mers that could be (mis) read as *k*mer *x_l_*,

This setup lends itself to maximum likelihood estimation via the EM algorithm [19]. The update equations are adapted from [16] using a different error model and are given as follows:

The expectations of hidden data *Y_lm_* obtained in the E step are

The M step yields maximum likelihood estimates

Notice the estimated expected number of attempts to read *k*mer *x_l_* is *T_l_* = *ŝ_l_N*(*L* – *k* + 1), directly proportional to *ŝ_l_* and sitting on the same scale as *Y_l_.* In fact, by observing the E step is unchanged and the log likelihoood *l*(***s** |****Y***) is computed up to an additive constant when *s_l_* is replaced with *T_l_*, we use the EM algorithm to compute *T_l_* directly. For inference, we apply the threshold to estimates ***T*** = (*T*_1_,…,*T_|R^k^|_*) rather than ***ŝ***, to more easily compare our method with thresholding on ***Y****.* The algorithm is initialized by setting *T_l_* =*Y_l_* and iterating until the log likelihood converges.

### Error detection and correction

Error detection, in practice, requires a method to choose a threshold *M* that minimizes the number of wrong decisions when classifying *k*mers as erroneous or not. We discuss a model-free method for estimating the threshold *M* in the Appendix, but no results presented in this paper use estimated thresholds.

To correct errors, consider each of the nucleotides in a read *r*. Each nucleotide appears in at least one and up to *k k*mers. Suppose the nucleotide at position 1 ≤ *i* ≤ *L* of the read appears at position 1 ≤ *t* ≤ *k* of *k*mer *x_l_.* The probability that the true nucleotide at position *t* was *b* prior to possible misread is

where estimates *T_m_* are substituted for the unknown *α_m_*. Since multiple overlapping *k*mers provide non-independent information about the base at position *i*, we average across available *t* to obtain distribution *p_i_*(*b*)*.* If argmax*_b_p_i_*(*b*) ≠ *r*[*i* : *i*], then we declare nucleotide *r*[*i* : *i*] misread and correct it to argmax*_b_p_i_*(*b*). To limit computations, we apply this method to reads likely to contain at least one erroneous *k*mer, as identified with a liberal threshold *M.*

## Results and discussion

### Dataset preparation

In order to test our model, we compiled various simulated and real datasets (Table 1). The datasets are classified into the following types (Table 1, column 2). Type 1 are simulated Illumina reads from (a) synthetically constructed genomes embedded with various types of non-overlapping repeats, and (b) previously sequenced genomes known to be rich in repeats. Type 2 are actual Illumina reads from a previously sequenced genome with a low degree of repetition.

**Table 1:** Experimental datasets

Dataset	Type	Reference genome	Genome length	Repeats	Repeat Types (length, multiplicity)	*C*	Number of reads
*D*1	1(a)	-	1M	20%	(1000, 200)	80x	2.2M
*D*2	1(a)	-	1M	50%	(500, 400), (1500, 200)	80x	2.2M
*D*3	1(a)	-	1M	80%	(500, 400), (1500, 200)	80x	2.2M
					(3000, 100)		
*D*4	1(b)	*N. meningitidis*	2.1M	-	-	80x	4.8M
*D*5	1(b)	Maize	418K	-	-	80x	0.92M
*D*6	2	*E. coli*	4.6M	-	-	160x	20.7M

‘-’ denotes the information that is not quantified; K: thousand; M: million.

#### Reference genome preparation

The reference genomes of type 1(a) were initially generated using the nucleotide distribution of a piece of B73 maize genome (A: 28% C:23% G: 22% T: 27%). Then, repeat regions of different lengths and multiplicities (Table 1, column 6) derived from the same nucleotide distribution were embedded at random locations in these reference genomes. The reference genome *N. meningitidis* (NC_013016) of *D*4 is known to be a small, repeat rich, viral genome. The maize genome is known to contain up to 80% repeats and only the relatively unique regions have been fully assembled. Hence, we concatenated the first 20 contigs from Chromosome 1 of the B73 assembly, and removed all non-ACGT characters to form the reference genome of *D*5.

#### Short read preparation

The simulated Illumina reads (type 1) were produced by first estimating an error distribution from a real Illumina short read dataset, then simulating uniformly distributed reads of the reference genomes with these error rates. We used the RMAP software [9] to map Illumina data (Sequence Read Archive ID: SRX000429) to the reference genome *E. coli str. K-12* allowing up to three mismatches. We were able to map 98.5% of reads; this percent is increased to 99.1% by allowing up to ten mismatches. However, allowing more mismatches increases the chance of a mismapped read since reads are only 36bp, and typically, mapping software can work at full sensitivity for up to two mismatches. Unmapped reads were discarded, and all remaining reads were assumed correctly mapped. By comparing the mapped reads to the reference genome, we estimated *L* 4 × 4 misread probability matrices ***M*** = (*M*_1_,M_2_,…,*M_L_*), where *L* is the read length and each entry (*α*, *β*) (*α*, *β* ∈ {*A*, *C*, *G*, *T*}) in misread probability matrix *M_i_* (1 ≤ *i* ≤ *L*) is the probability a nucleotide *α* on the reference genome is (mis)read as *β* at position *i* in the read. This is calculated as the total number of times *α* is read to be *β* at position *i* among all mapped reads, divided by the number of times the corresponding position of the reference genome is *α.* Finally, we simulated Illumina sequencing to generate *N* reads by applying ***M*** to *N* uniformly distributed *L*-substrings in the reference genome.

#### Rationale

Simulated data are essential because highly repetitive genomic regions, for which our error model is designed, are often masked prior to assembly. Even when assembly can be done, accurate mapping of sequenced reads back to the assembly is difficult when genomes are repetitive [20]. Under these conditions, only simulation can provide unambiguous error information. Type 1(a) datasets were prepared such that they emulate repeat content ranging from a microbial genome with low repeats to a highly repetitive plant genome. However, to inject reality wherever possible, the reference genomes of Type 1(b) were selected from the previous assemblies. Lastly, the type 2 dataset demonstrated the applicability of our model to real, although non-repetitive, real read data.

### Error detection and correction results

Our model accommodates sequencing errors via the misread probabilities *p_e_*(*x_m_*, *x_l_*) between any two *k*mers *x_m_* and *x_l_.* To calculate *p_e_*(*x_m_*,*x_l_*), we need to specify the position specific misread probabilities, *q_i_*(α, *β*), 1 ≤ *i* ≤ *k*, *α*, *β* ∈ {*A*, *C*, *G*, *T*}, for each position of a *k*mer. Ideally, we would set *q_i_*(·, ·) to match the errors in the current dataset inferred from reads in the control lane [11,17]. When such information is not available, we can rely on information derived from other read data generated on the same platform. In the worst case, we can use the simple error model of Eq. (1), which only requires specification of the average error rate *p_e_.*

Based on these choices, we tested our datasets using four types of sequencing error (misread) distributions: tIED, wIED, tUED, and wUED (defined below). Our simulation procedure introduced errors according to the misread probability matrices ***M*** estimated from dataset SRX000429, so the true error distribution, tIED, was obtained by estimating *q_i_*(·, ·) from the same dataset SRX000429. The estimation procedure is similar to the one used for estimating ***M*** (defined in the previous section), except each read is decomposed into *L* – *k* + 1 *k*mers and the count of each type of misread nucleotide at each *k*mer position is determined. (Note, the same nucleotide contributes counts in up to *k* distinct *k*mers.) Since, the estimated *q_i_*(·, ·) represent fewer parameters than ***M***, *q_i_*(·, ·) only approximates the true misread probability matrices ***M***, which themselves only approximate true read errors. The *wrong* Illumina error distribution, wIED, is the situation encountered when Illumina data are only available from a different experiment (and often different lab). To emulate this case, we derived a second set of error probabilities *q_i_*(·, ·) from Illumina reads of *Acinetobacter sp. ADP1* (Short Read Archive acc. SRX001814, 17.7M reads of 36bp length). The error rates differ at *k*mer position *i* = 11 (Table 2) and others (not shown) in the *E. coli* and *A. sp. ADP1* short read datasets, demonstrating that wIED is indeed the *wrong* error distribution. Finally, in the absence of detailed error information, we can use the uniform error distribution with constant error probability *p_e_*. When the average error rate *p_e_* = 0.006 is estimated from dataset SRX000429, the error distribution is the true uniform error distribution (tUED). When the error rate is *over*estimated at *p_e_* = 0.02, above the published rate of 0.01–0.015 [21], it is the *wrong* uniform error distribution, wUED.

The same measures as in [14] are used for evaluation, where a false positive (FP) denotes an error free *k*mer has been considered as erroneous and a false negative (FN) denotes an unidentified erroneous *k*mer. Table 3 reports the minimum number of wrong predictions (WPs), FP+FN, achieved by applying optimum thresholds on observed ***Y*** , used by existing methods, or by applying thresholds on the estimated number of attempts to read ***T***, used in our method. The results of our method are shown for the four types of error distributions in columns tIED, wIED, tUED, and wUED. Bolded entries indicate where lower minima were achieved with our method compared to the standard method. Given the *true* error distribution, our method committed over 95% *fewer* WPs for all datasets except *D*6, where our method still managed 7% fewer WPs. Interestingly, using the wrong Illumina error distribution (column wIED) achieved at least 33% fewer WPs in all repetitive genomes except *D*4, where our wIED method performed about on par with applying the threshold on ***Y*** . The minimum WPs achieved by the true uniform error model tUED are two- to three-fold smaller than the corresponding values in column *Y* . However, using elevated error rate *p_e_* = 0.02 led to higher minimum WPs, except in dataset *D*3, the most highly repetitive simulated genome.

**Figure 2:**
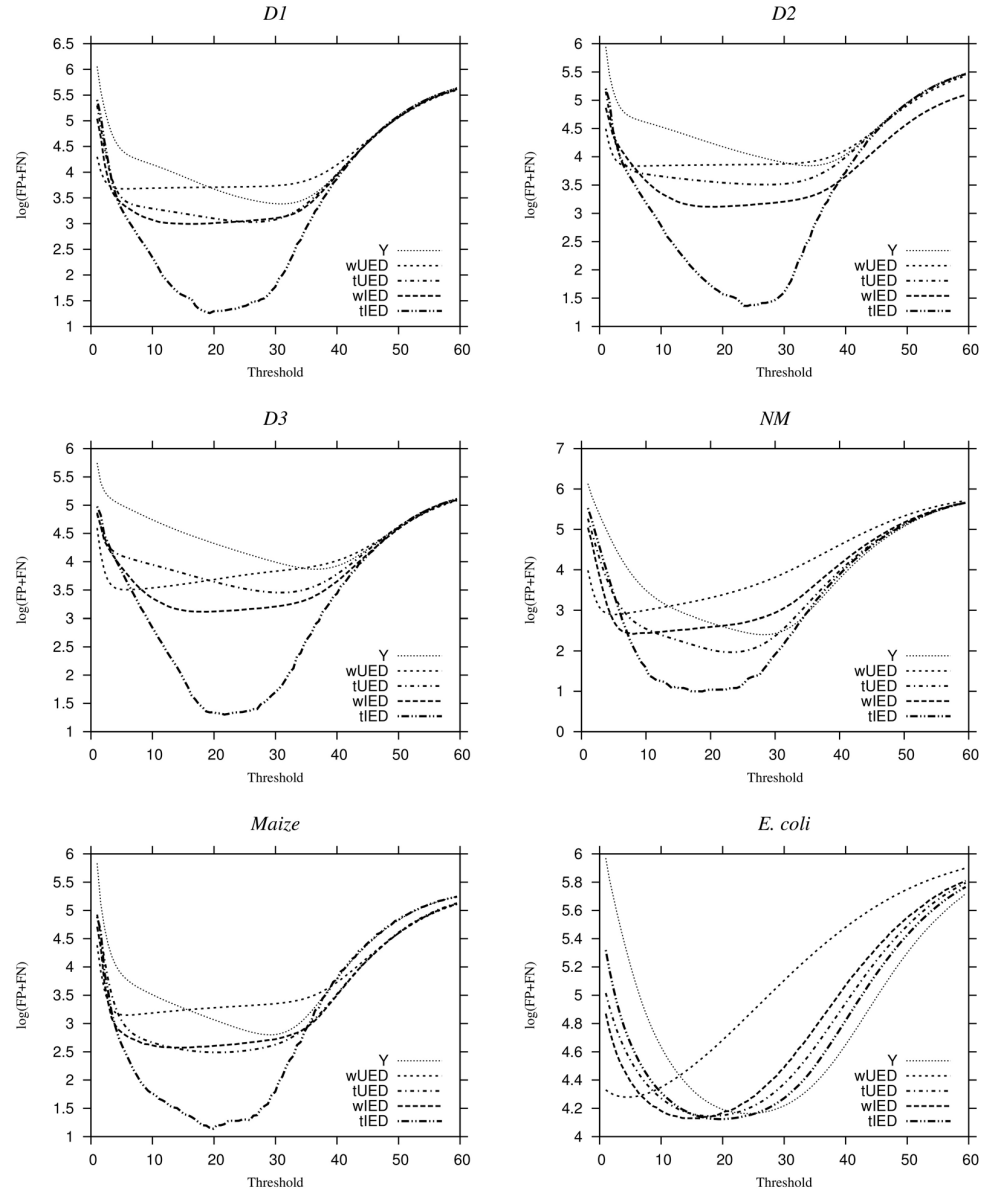
**Plots of log(FP + FN) vs. threshold for all datasets.** In each plot, we compare the results by applying thresholds to ***Y*** and to ***T*** estimated by our model using the tIED, wIED, tUED and wUED error distributions.

Even though we presented a method to choose the threshold value (see Appendix), it is not possible for any method to guarantee the optimal threshold. Ideally, the error detection methods should be relatively insensitive to choice of threshold. To compare methods across many thresholds, we plot log(FP + FN), with respect to a wide range of thresholds (Fig. 2). The plot in every case resembles a U-shape since many error *k*mers are missed (FN) when the threshold is too low and many correct *k*mers are declared errors (FP) when the threshold is too high. Our method achieved fewer WPs for datasets *D*1,*D*2,*D*3 and *Maize* with error distributions tIED, wIED, and tUED at *all* thresholds. The improvement in error detection increased with the degree of repetition, seen in simulations *D*1 to *D*3 and also in comparing *N. meningitidinis* and *Maize*. Our method tended to flatten the bottom of the U-shape such that a wider range of thresholds often beat even the minimum error obtained under ***Y*** thresholding. In all datasets, our method often shifted the U-shape leftward, such that for very small thresholds, our WPs were far less than the ***Y*** -based methods, regardless of the error distribution used. As the threshold increases, WPs for all methods eventually converge to the same constant, where all *k*mers are considered erroneous. For moderately large thresholds, our method sometimes resulted in higher WPs, especially for the wrong error distribution wUED, and sometimes wIED, and genomes with a low degree of repeats.

**Table 2:** Estimated error probabilities *q_i_*(·, ·), position *i* = 11

*E. coli str. K-12 substr.*						*Acinetobacter sp. ADP1*				
		
×10^–2^	A	C	G	T		×10^–2^	A	C	G	T
		
A	98.96	0.63	0.18	0.23		A	96.18	2.53	0.19	1.10
C	0.15	99.60	0.10	0.15		C	0.20	99.32	0.08	0.40
G	0.05	0.17	99.25	0.53		G	0.12	0.30	97.60	1.98
T	0.05	0.19	0.18	99.58		T	0.09	0.18	0.13	99.60

**Table 3:** A comparison of minimum error rates.

Data	Minimum (FP + FN) Value				

	*Y*	tIED	wIED	tUED	wUED
*D1*	2212	**18**	**984**	**1020**	4648
*D2*	6392	**23**	**1300**	**3150**	6729
*D3*	6809	**19**	**1300**	**2696**	**3124**
*D4*	216	**10**	236	**80**	719
*D5*	552	**14**	**373**	**297**	1346
*D6*	14236	**13275**	**13441**	**13671**	18793

A comparison of the minimum number of wrong predictions achieved by applying optimum thresholds to observed occurrences ***Y*** , and our model with each of the error distributions tested. Bold numbers indicate that our model outperforms.

REDEEM misclassified the fewest *k*mers when using the “true” error model, but even in our simulations, there was a mismatch between the simulated errors and the estimated “true” error model. The position-specific error probabilities used to compute *k*mer misread probabilities are not the true error probabilities that vary by position in the full length read. The difference is exacerbated as reads get longer relative to the *k*mer length. Since it would be possible to compute misread probabilities *p_e_*(*x_m_*, *x_l_*) using read-derived position probabilities, this mismatch between *k*mer and read errors can be eliminated with more sophisticated error models that account for the position of each *k*mer in the read. Since data to estimate the read error properties can be collected in parallel on a known, control genome, we contend that estimating the true error model is not an undue burden in practical applications [11,17]. Quality scores may also inform on errors [15] and could be incorporated in the REDEEM error model.

As discussed previously, only simulated data with different degrees of repeats can be utilized to measure error correction results for repeat-rich genomic regions due to the fact that mapping short reads from such regions uniquely to the reference genome, and the assembly of genomes with high repeat content, remain open problems. We compare our correction results with SHREC [12] and Reptile [13] using datasets *D*1, *D*2 and *D*3 with increasing degrees of repeat content. The results are shown in Table 4. To be self-contained, we reproduce the evaluation measures from [13]: A *True Positive* (TP) is any erroneous base that is changed to the true base, a *False Positive* (FP) is any true base changed wrongly, a *True Negative* (TN) is any true base left unchanged, and a *False Negative* (FN) is any erroneous base left unchanged. *Sensitivity* = TP / (TP + FN) and *Specificity* = TN / (TN + FP). *Gain* = (TP - FP) / (TP + FN) denotes the total percentage of erroneous bases removed from the dataset post-correction.

REDEEM is designed to specifically target error correction for repeat-rich genomes. While both SHREC and Reptile do not explicitly model the effect of repeats, the variable length suffixes captured by SHREC and different read decompositions explored by Reptile can provide richer and more precise information about errors. Currently, REDEEM does not utilize all such information. Therefore, in genomes with low repeat content, both SHREC and Reptile outperform. However, as the repetition within the genome increases, REDEEM significantly outperforms both methods by accounting for the repetition in the *k*mer neighborhood. Further experiments show that error correction results are affected mainly by the percentage of the length of the genome spanned by repeats, rather than repeat types and lengths. This can serve as a yardstick in deciding when to use REDEEM over conventional error correction. It is also possible to combine the features of a conventional error correction method such as Reptile with the explicit modeling of repeats as done in REDEEM to produce an error-correction method that is superior both when sampling low repeat and highly-repetitive genomes.

**Table 4:** Error correction results

Data	Method (*d*)	Sensitivity	Specificity	Gain	CPU Time(min)	Memory(GB)
*D*1	SHREC	81.2%	99.9%	80.3%	23.9	5.9
	Reptile	78.9%	99.9%	78.9%	0.6	0.19
	REDEEM	71.3%	99.9%	51.5%	114.1	2.5

*D*2	SHREC	54.0%	99.9%	52.7%	22.7	5.8
	Reptile	57.8%	99.9%	57.8%	0.5	0.16
	REDEEM	78.6%	99.9%	64.6%	72.7	1.6

*D*3	SHREC	29.3%	99.9%	26.7%	21.7	5.8
	Reptile	46.8%	99.9%	46.8%	0.5	0.13
	REDEEM	86.4%	99.9%	79.4%	31.2	0.63

All experiments were carried out on 3.16GHz Intel Xeon Processors; run time and memory usage of all three programs are shown in the last two columns in Table 4. As expected, the run time of REDEEM is longer due to the complexity of modeling repeats explicitly. The largest simulation, D6, took 120 minutes and 9 GB. No error detection/correction method except naÃ¯ve thresholding on observed counts yet scales to practical next-generation applications, but REDEEM is at least comparable to existing, non-repeat-aware methods.

## Conclusions

There have been some attempts to formally characterize repeats in genomes [22], but generally, the term “repeat” is used loosely in the literature, with meaning varying by context. In this paper, we consider *k*mer *x_l_* a repeat when its genomic occurrence *α_l_* is higher than what is expected in a random genomic sequence. Because genomes are not random, all genomes display some degree of repetition. Perhaps such cryptic repetition explains why we can achieve lower false prediction rates at optimal thresholds even on genomes like *E. coli*, which according to the *I_r_* measure of [22] is only somewhat repetitive.

In summary, we have presented a new method that improved error detection and correction when sampling repeat-rich genomic regions using next-generation sequencers. Important future work includes better models and algorithms to simultaneously estimate error parameters from the data, to consider variation in coverage along the genome, to speed up computations, and to handle larger datasets through better memory management.

## Competing interets

The authors declare that they have no competing interests.

## Authors contributions

XY and KD developed and implemented the model and algorithmic solutions. SA helped conceive of and coordinated the study. All authors contributed to the development and revision of the manuscript.

## Appendix

**Figure 3:**
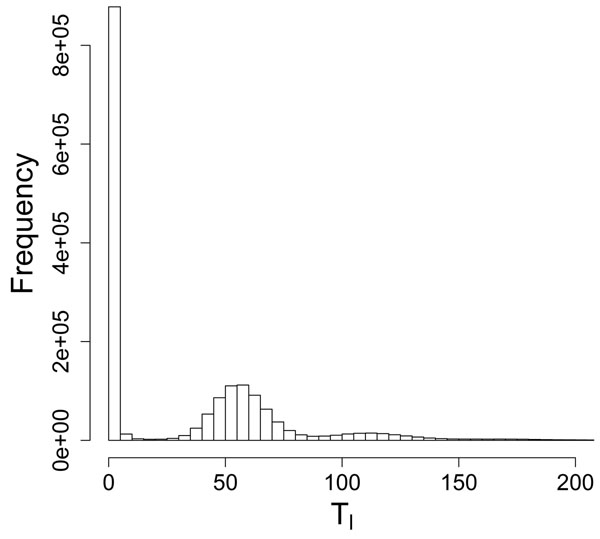
Histogram of estimated *T_l_* for E. coli dataset.

The true expected number of attempts to read *k*mer *x_l_*, , are constant multiples of the discrete genome occurrences *α_l_* ∈ {0,1,…,}, where the coverage-related constant  is unknown. The estimated *T_l_* vary from these true values because of sampling and estimation error. A histogram of estimated *T_l_* (see Fig. 3 for the *E. coli* dataset) thus reveals peaks corresponding to *α_l_* = 0, *α_l_* = 1, and *α_l_* = 2*.* The constant multiple is about 57, which can also be verified from Table 1. The *k*mers with *T_l_* near 0 have no occurrences in the genome. One approach to model multi-modal distributions, such as that of Fig. 3, is to use a mixture model. Then, erroneous *k*mers would be those derived from the mixture component corresponding to the first mode. We propose mixture distribution(2)

where the mixing probabilities π_0_,…,π***_G_***_+1_ sum to 1. The first component *f*(*α*, *β*) of the mixture is a Gamma distribution, corresponding to the erroneous *k*mers. The second through (***G*** + 1)th components are a series of normal distributions fitting the subsequent peaks for *α_l_* = 1,2,…,***G****.* The last component is a uniform distribution over the observed range of *T_l_*. We use the uniform distribution to account for the few *k*mers with large *α_l_* > ***G****.* One particular parameterization of the normal components is justified as follows. Under the assumption of reads distributed uniformly throughout the genome, the true *T_l_* are Poisson random variables, where the mean depends on the identity of *k*mer *x_l_* and the error model. Rather than model the errors again, we hypothesize these means are Gamma random deviates. Then, *T_l_* follows a Negative Binomial distribution [24], with means *µ_g_* = *gµp*/(1 – *p*) and variances  when *α_l_* = *g* ∈ {1,…,***G***}*.* Finally, by the Central Limit Theorem, the Negative Binomial is well-approximated by the Normal distribution with matching means and variances when the coverage-related parameter  is large.

We can obtain the maximum likelihood estimate of the parameter vector *θ* = (π_0_,…,*π****_G_***_+1_, *α*, *β*, *µ*,*p*) using another EM algorithm. Let *Z_lg_*, *l ∈* {1,…,|*R^k^*|}, *g ∈* {0,…,***G*** + 1} indicate if *k*mer *x_l_* is in group *g.* Then, the complete log likelihood for the proposed mixture model (Eq. 2) is

Let  be the probability *x_l_* belongs in group *g* given the current estimate  of all model parameters. For computational efficiency, at each iteration, we first compute

the first three for all *g* = 0,…,***G*** + 1. Here, *N_g_* is the number of *k*mers in group *g*, *T* is the number of attempts to read a random *k*mer, and *Z_g_* indicates if this *k*mer is in group *g.* Then, the update equations for the parameters are

where  is found as the root of

given  above. This and the second equation are implicit functions for  and  that can be solved using any one-dimensional root finder. To choose the best number of groups , we compute and minimize the BIC [23] over a range of plausible ***G****.* Members of the gamma component that represent *k*mers *not* present in the genome are identified as those with .
